# A prospective, randomised, placebo-controlled, double-masked, three-armed, multicentre phase II/III trial for the Study of a Topical Treatment of Ischaemic Central Retinal Vein Occlusion to Prevent Neovascular Glaucoma – the STRONG study: study protocol for a randomised controlled trial

**DOI:** 10.1186/s13063-017-1861-3

**Published:** 2017-03-16

**Authors:** Katrin Lorenz, Yvonne Scheller, Katharina Bell, Franz Grus, Katharina A. Ponto, Felix Bock, Claus Cursiefen, Jens Flach, Marta Gehring, Tunde Peto, Rufino Silva, Yossi Tal, Norbert Pfeiffer

**Affiliations:** 1 Department of Ophthalmology, University Medical Center, Johannes Gutenberg-University Mainz, Langenbeckstr. 1, D-55131 Mainz, Germany; 2 Center for Thrombosis and Hemostasis, University Medical Center, Johannes Gutenberg-University Mainz, Langenbeckstr. 1, 55131 Mainz, Germany; 3 Department of Ophthalmology, University of Cologne, Kerpener Str. 62, 50924 Cologne, Germany; 4 Bundesverband Glaukom-Selbsthilfe e.V., Märkische Str. 61, 44141 Dortmund, Germany; 5 Gene Signal International SA, EPFL Innovation Park-A, 1015 Lausanne, Switzerland; 6 NIHR Biomedical Research Centre at Moorfields Eye Hospital NHS Foundation Trust and UCL Institute of Ophthalmology, London, UK; 7 Faculty of Medicine, University of Coimbra (FMUC), Azinhaga de Santa Comba, Celas, 3000-075 Coimbra, Portugal; 8 Department of Ophthalmology, Coimbra Hospital and University Center (CHUC), Praceta Prof. Mota Pinto, 3000-075 Coimbra, Portugal; 9 Association for Innovation and Biomedical Research on Light and Image (AIBILI), Azinhaga de Santa Comba, Celas, 3000-548 Coimbra, Portugal; 10 TechnoSTAT Ltd., 34 Jerusalem Rd., Raanana, 4350108 Israel

**Keywords:** Neovascular glaucoma, Neovascularisation, Ischaemic central retinal vein occlusion, Intraocular pressure, Aganirsen, Placebo, Topical treatment, Orphan disease

## Abstract

**Background:**

Neovascular glaucoma (NVG) is rare, comprising only 3.9% of all glaucoma cases. The most common cause of NVG is ischaemic central retinal vein occlusion (iCRVO). NVG frequently results in blindness and painful end-stage glaucomatous damage leading to the need for enucleation. Currently, there is no preventive therapy for NVG following iCRVO. Rescue treatments have severe drawbacks. Accordingly, there is a great need for preventing the often visually devastating outcomes of NVG. The STRONG study is designed to test whether the topically active anti-angiogenic agent aganirsen is able to inhibit the formation of neovascularisation leading to the development of secondary NVG in eyes with iCRVO. At the same time, STRONG will provide important information on the natural course of iCRVO and NVG in a large and well-characterised cohort of such patients.

**Methods/design:**

This protocol describes a phase II/III, prospective, randomised, placebo-controlled, double-masked, three-armed multicentre study for the investigation of aganirsen, a new topical treatment for iCRVO in order to prevent NVG. The study will evaluate the efficacy of two different doses of this newly developed antisense oligonucleotide formulated in an eye emulsion to avoid new vessel formation by blocking insulin receptor substrate-1 (IRS)-1. This leads to subsequent down-regulation of both angiogenic as well as proinflammatory growth factors such as vascular endothelial growth factor (VEGF) and tumour necrosis factor (TNF). Eligible patients (*n* = 333) will be treated with topical aganirsen or placebo for a period of 24 weeks. They will also be invited to participate in substudies involving analysis of gonioscopic images, detection of biomarkers for NVG and risk factors for iCRVO.

**Discussion:**

The STRONG study has the potential to offer a new treatment modality for patients suffering from iCRVO with a high risk of developing NVG. The topical administration can reduce patients’ burden and risk related to rescue treatment, such as destructive laser treatment or enucleation, but requires a high level of patient compliance.

**Trial registration:**

EudraCT: 2014-000239-18; ClinicalTrials.gov, ID: NCT02947867. (Registered on 15 October 2016); see also http://strong-nvg.com.

**Electronic supplementary material:**

The online version of this article (doi:10.1186/s13063-017-1861-3) contains supplementary material, which is available to authorized users.

## Background

Glaucoma is a group of conditions characterised by progressive optic nerve degeneration and loss of visual function, ultimately resulting in blindness. In contrast to very frequent types of glaucoma, secondary neovascular glaucoma (NVG) is rare at approximately 3.9% of all glaucoma cases. NVG is very aggressive, the prognosis is poor, and early, aggressive treatment is required to prevent blindness and painful end-stage glaucoma with irreversible loss of the eye. One common cause of NVG is ischaemic central retinal vein occlusion (iCRVO) [1–4]. iCRVO is a retinal pathology resulting in hypoxia, which leads to the expression of insulin receptor substrate-1 (IRS-1). IRS-1 in turn increases VEGF expression further along in the cascade [5] resulting in the pathogenic development of new vessels in the anterior and/or posterior segment of the eye. This new vessel formation can build an irreversible fibrovascular membrane over the trabecular meshwork and iris, leading to an obstruction of the trabecular meshwork and a clinically relevant increase in intraocular pressure (IOP) [4].

The study drug aganirsen is a 25-mer phosphorothioate antisense oligonucleotide of 8035 Da that inhibits new vessel formation by blocking IRS-1 and thus VEGF and tumour necrosis factor-alpha (TNF-alpha) [6, 7]. Aganirsen eye drops (saline solution) are safe at the maximum dose tested of 430 μg/day, which is equivalent to 10-fold and 5-fold the doses planned by the proposed study. Aganirsen eye drops have previously been shown in both phase II and III clinical trials to significantly inhibit corneal neovascularisation and to be well tolerated [8, 9]. Because the STRONG study planned by this consortium will be undertaken with a novel galenic formulation (emulsion) instead of saline eye drops, a comparative safety bridge study was performed for the two formulations (eye drops for corneal neovascularisation, and emulsion for prevention of NVG). No safety concerns occurred during the study.

Currently, reduction of IOP remains the focus of all therapeutic approaches for the majority of glaucoma patients, including those with NVG. For NVG, however, IOP-lowering medications are moderately helpful because outflow is obstructed and the accompanying inflammation and proliferative stimulus lead to the very poor success rate of fistulating glaucoma surgery. Currently, the common therapy for neovascularisation is pan-retinal photocoagulation (PRP), although additional anti-VEGF intravitreal injections have become more and more common for the therapy of neovascularisation. Thermic laser coagulation of the retina by PRP is strongly advocated and practiced widely for the management of iCRVO if new vessels have already formed [10, 11]. However, no definite guidelines exist regarding the exact indication and timing of PRP. As an alternative method of selective retinal ablation, pan-retinal cryotherapy may be performed on the peripheral fundus if the ocular media are too hazy for laser application.

There is a clear need to widen the existing therapeutic options for the treatment of iCRVO to prevent neovascularisation and NVG, with a specific focus on noninvasive therapeutic options aimed at prevention or early management respectively of the neovascular component of iCRVO. Hence, it is expected that aganirsen will benefit patients with iCRVO by interfering at an early stage in the neovascularisation process. Understanding the natural course of NVG, including biomarker and risk factor analysis, will further support the evaluation of new diagnostic and therapeutic interventions. These results could help to make early NVG diagnosis independently of an increased IOP by analysis of body fluids.

The primary objective of this multicentre randomised controlled study is to assess the efficacy and safety of aganirsen relative to placebo in preventing NVG in patients with iCRVO. We will evaluate the appearance of neovascularisation and the rise in IOP after 24 weeks (co-primary endpoint). We hypothesise that each of the two tested aganirsen doses will be superior to the placebo in preventing NVG at week 24. This study will also evaluate the effective dose of aganirsen.

The secondary objectives are to evaluate the efficacy of two aganirsen doses, to assess the aganirsen efficacy relative to placebo on time to, and intensity of, additional interventions, such as PRP or cryotherapy, to compare the effect of aganirsen and placebo on health outcome and quality of life (QoL) and to confirm a positive safety profile of aganirsen.

The aim of the imaging substudy is to develop an objective quantification of neovascularisation in the anterior part of the eye. For the biomarker substudy, the aim is to find possible biomarkers for iCRVO and NVG in tears and serum. The aim of the risk factor substudy is to identify potential novel risk factors for iCRVO and to evaluate if some of those parameters can be useful to differentiate patients who are at a higher risk of developing NVG.

## Methods/design

Trial sponsor: Gene Signal SA, Genopole Biopark, Evry (Paris), France. Coordinating centre: University Medical Center at Johannes-Gutenberg University Mainz, Germany, Department of Ophthalmology. The study is funded by the European Commission 7th Framework Programme (Grant number 305321). This funding source had no role in the design of this study and will not have any role during its execution, analysis, data interpretation and dissemination of study results. The dissemination of study results is scheduled in a publication master plan and rights are organised in a consortium agreement. A Scientific Advisory Board and a Data Safety Monitoring Board will oversee the trial.

**Fig. 1:**
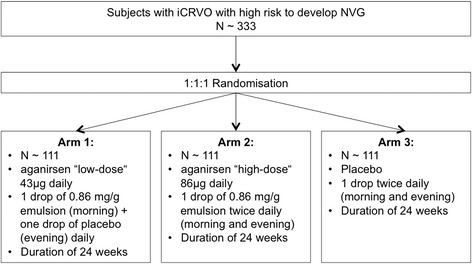
STRONG trial design

The STRONG trial is a phase II/III, prospective, 1:1:1 randomised, placebo-controlled, double-masked, three-armed multicentre trial for the study of the topical treatment of iCRVO to prevent NVG with a newly developed eye emulsion. It plans to enrol up to 333 patients suffering from a primary iCRVO or a conversion to iCRVO for no longer than 4 weeks in approximately 35 clinical sites in eight countries across Europe (Germany, France, Italy, Great Britain, Portugal, Spain and Hungary and Belgium; for a detailed list see www.strong-nvg.com). Trial staff will be trained and will follow standard operating procedures for the specific assessments and data collection. One eye per subject will be enrolled in the study. The study drug aganirsen will be administered as a topical emulsion in two different dosages and will be compared with a placebo group (Fig. 1).

**Fig. 2:**
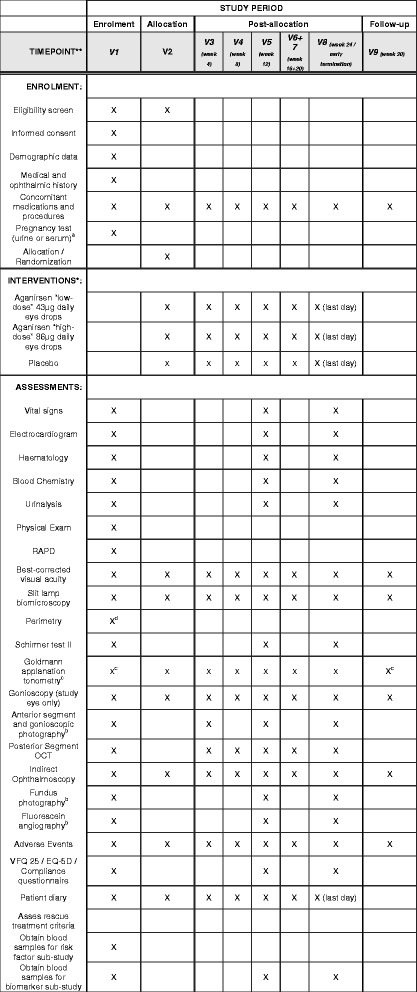
Standard Protocol Items: Recommendations for Interventional Trials (SPIRIT) figure

Each patient who has signed the Informed Consent Form is monitored for a period of 30 weeks (24-week treatment phase and 6-week safety follow-up) during which patients will attend nine visits (examination schedule in Table 1; see also Fig. 2. (Additional file 1). A computer-based centralised randomisation scheme for all study sites will employ a 1:1:1 treatment assignment ratio (main study) using block randomisation with sequential numbering for each specific trial site. Subjects, investigators, site personnel, monitoring staff, data management and other sponsor personnel, excluding the Clinical Supplies Unit, will be masked to subjects’ assignments. Only in the case of a serious adverse event (SAE), when the masked information is urgently needed for the treatment of the subject, will treatment allocation be revealed.

**Table 1:** Examination schedule

Protocol activity	Screening	Baseline	Week 4	Week 8	Week 12	Week 16 + 20	Week 24/early termination	Week 30 (follow-up)
	V1	V2	V3	V4	V5	V6 + 7	V8	V9
Informed consent	X							
Demographic data (age, gender)	X							
Medical and ophthalmic history	X							
Concomitant medications and procedures	X	X	X	X	X	X	X	X
Adverse events	X	X	X	X	X	X	X	X
NEI-VFQ-25/EQ-5D/ Compliance questionnaires		X			X		X	
Vital signs (blood pressure/pulse)	X				X		X	
Electrocardiogram	X						X	
Haematology	X				X		X	
Blood chemistry	X				X		X	
Urinalysis	X				X		X	
Obtain blood sample for risk factor substudy	X							
Obtain blood sample for biomarker substudy	X				X		X	
Pregnancy test (urine or serum)^a^	X							
Physical exam	X							
Best-corrected visual acuity (BCVA) (ETDRS)	X	X	X	X	X	X	X	X
RAPD	X							
Slit lamp biomicroscopy	X	X	X	X	X	X	X	X
Goldmann perimetry/semi-automatic kinetic perimetry	X^d^							
Schirmer test II (biomarker substudy)	X				X		X	
Goldmann applanation tonometry (±1 h)	X^c^	X	X	X	X	X	X	X^c^
Gonioscopy (study eye only)	X	X	X	X	X	X	X	X
Anterior segment and gonioscopic photography^b^	X		X		X		X	
Posterior segment OCT (SD-OCT)	X		X	X	X	X	X	
Indirect ophthalmoscopy	X	X	X	X	X	X	X	X
Fundus photography^b^	X				X		X	
Fluorescein angiography^b^	X				X		X	
Review inclusion/exclusion criteria	X	X						
Randomisation		X						
Administer study treatment (aganirsen or placebo)		X						
Drug dispensing/return and bottle weight		X	X	X	X	X	X	
Patient diary dispensing/return		X	X	X	X	X	X	
Assess rescue treatment criteria			X	X	X	X	X	X

*BCVA* best-corrected visual acuity, *EDTRS* Early Treatment Diabetic Retinopathy Study, *EQ-5D* EuroQoL five dimensions questionnaire, *IEC*, *Independent Ethics Committee*, *IOP* intraocular pressure, *NEI*-*VFQ-25* National Eye Institute Visual Function questionnaire 25, *RAPD relative afferent pupillary defect*, *SD-OCT* spectral domain optical coherence tomography

^a^Pregnancy tests may also be repeated as per request of IECs or if required by local regulations. Only applicable for women of childbearing potential

^b^Optional at all other visits: only if neovascularisation is suspected in slit lamp examination, gonioscopy or indirect ophthalmoscopy

^c^IOP measurement by Goldmann applanation tonometry can be performed at any time of the day at the screening visit, V9 and at an unscheduled visit. The time (±1 h) of IOP measurement at the baseline visit is mandatory for all other visits (V3–V8)

^d^Perimetry can be skipped if eligibility is fulfilled by other criteria

The two study groups will be treated with aganirsen ‘low-dose’ (43 μg per day) or ‘high-dose’ (86 μg per day). The control group will receive placebo, as there is no labelled treatment for iCRVO to prevent NVG. The placebo is optically identical to the aganirsen emulsion.

Images of the anterior segment and gonioscopic photos of all patients participating in the STRONG study will be collected and evaluated for neovascularisation within the imaging substudy. Tears and serum samples will be collected during the biomarker substudy from 200 patients to evaluate possible markers for both iCRVO and NVG. The results will be compared with results of healthy subjects already available in a German database. Thirty-three eligible patients will be included in the risk factor substudy across all participating trial sites. At UMC Mainz, 33 age- and gender-matched healthy controls will be recruited. Blood samples will be collected to analyse laboratory parameters for coagulation and fibrinolysis and genetic factors to identify risk factors for iCRVO.

Protocol assistance at the European Medicines Agency (EMA) has taken place to define patients with a high risk for developing NVG. The inclusion criteria must be fulfilled at both the screening and baseline visits.

### Inclusion criteria

- Primary iCRVO or conversion to iCRVO for no longer than 4 weeks in the study eye
- Best-corrected visual acuity (BCVA) ETDRS letter score <35 letters (<20/200 Snellen equivalent) in the study eye
- At least a 10-disc area of retinal capillary obliteration on fluorescein fundus angiography in the study eye (central fundus: macular area as defined by the optic disc and the arcades, an approximate 6000-μm circle around the fovea) and/or large, confluent retinal haemorrhages in the study eye
- IOP in the study eye ≤21 mmHg
- Male or female adults ≥18 years
- For men and women of childbearing potential, willingness to utilise adequate contraception and not become pregnant (or have their partner become pregnant) during the full course of the study. Adequate contraceptive measures include (oral) hormonal contraceptives (stable use for two or more cycles prior to screening) and other prescription pharmaceutical contraceptives, intrauterine device, bilateral tubal ligation, vasectomy, condom, or diaphragm plus contraceptive sponge, foam, or jelly
- Willing, committed and able to return for all clinic visits and complete all study-related procedures. A written informed consent must be provided before any study-related procedure can be performed

Additionally, at least four out of six criteria must be fulfilled:

1. A relative afferent pupillary defect (with a normal fellow eye)
2. At least ten cotton-wool spots in the study eye
3. Venous tortuosity in the study eye
4. Peripheral visual field defects corresponding to ischaemia: dense scotomas in areas of capillary nonperfusion in the study eye. Patient cannot see the I-2e target and has a defective or absent I-4e isopter (Goldmann perimeter or semiautomatic kinetic methods (Humphrey or Octopus visual field analyser), targets I-2e, I-4e, V-4e)
5. Engorged vessels on the iris and/or chamber angle in the study eye
6. Detectable anterior chamber flare in the study eye

### Exclusion criteria

1. Ocular conditions with a worse prognosis in the fellow eye than in the study eye
2. Primary or secondary glaucoma in the study eye
3. Ocular anti-VEGF treatment:
  - Any anti-VEGF ranibizumab or bevacizumab treatment in the study eye in the last 45 days before the screening visit
  - Any anti-VEGF aflibercept treatment in the study eye in the last 90 days before the screening visit
  - Anti-VEGF treatment in the fellow eye during the trial
4. Any prior or concomitant use of systemic anti-VEGF products
5. Previous use of intraocular corticosteroids in the study eye at any time or use of periocular corticosteroids in the study eye within 90 days prior to the screening visit
6. Previous use of intraocular or periocular corticosteroids in the fellow eye within 90 days prior to the screening visit
7. History of idiopathic or autoimmune uveitis in either eye
8. Presence of anterior segment neovascularisation (neovascularisation of the angle (NVA) or neovascularisation of the iris (NVI)), neovascularisation of the disc (NVD) or neovascularisation elsewhere (NVE) in the study eye
9. Previous PRP in the study eye
10. Previous cyclocryocoagulation in the study eye
11. Pregnancy and lactation
12. Any abnormality in the study eye preventing reliable applanation tonometry
13. Intraocular surgery (other than intravitreal anti-VEGF treatment) or laser treatment in the study eye within the last 90 days before the screening visit
14. Evidence at examination of infectious blepharitis, keratitis, scleritis, or conjunctivitis in either eye, or current treatment for serious systemic infection
15. Any ocular disorders in the study eye that, in the opinion of the investigator, might confound the interpretation of the study results or any other reason that, in the opinion of the investigator, precludes the subject from participating in this study
16. Inability to obtain fundus photographs or fluorescein angiograms of sufficient quality to be analysed
17. Allergy to fluorescein
18. History of hypersensitivity to the study drug or to any drug with a similar chemical structure or to any excipient present in the pharmaceutical form of the investigational medicinal products
19. History of other disease, metabolic dysfunction, physical examination finding, or clinical laboratory finding giving reasonable suspicion of a disease or condition that, in the opinion of the investigator, contraindicates the use of an investigational drug, might affect interpretation of the results of the study, or renders the subject at high risk from treatment complications
20. History of breast cancer
21. Medical or psychological condition that would not permit completion of the trial or signing of an informed consent
22. Participation in other clinical trials during the present clinical trial or within the last 4 weeks

For the risk factor substudy, biomarker and imaging substudy, the same inclusion and exclusion criteria must be met by the patients.

Additional exclusion criteria were defined for the eligibility of patients for the risk factor substudy: age 65 years or older, renal insufficiency and acute malignant or inflammatory reaction (systemic or local).

The co-primary efficacy endpoints combine a neovascularisation component: presence of anterior segment neovascularisation (NVI or NVA), NVD and/or NVE in the study eye requiring PRP rescue treatment or cryotherapy rescue treatment up to week 24 scored dichotomously (yes/no) and an IOP component: percentage change (≥20%) in IOP compared to baseline and >21 mmHg in the study eye scored ‘failure’, while otherwise ‘success’.

The secondary endpoints of the trial are the time of development of secondary NVG in the study eye up to week 24 (in case aganirsen does not totally inhibit but slows down the development of NVG); the time of development of anterior segment neovascularisation (NVI or NVA); NVD or NVE in the study eye requiring PRP or cryotherapy up to week 24; the development of changes in BCVA (ETDRS letter score) in the study eye up to week 24 (from baseline); the development of needed additional laser treatments and re-treatments in the study eye at up to week 24 including the intensity of the needed additional treatment; the development of changes in the size of retinal nonperfusion areas in the study eye up to week 24 in comparison with the baseline; the development of changes in the National Eye Institute Visual Function questionnaire 25 (NEI-VFQ-25) health questionnaire total score at week 24 in comparison with the baseline; the development of changes in the EuroQoL five dimensions questionnaire (EQ-5D) health questionnaire (validated questionnaires) score at week 24 in comparison with baseline; absolute change from baseline in central retinal thickness in the study eye, assessed by spectral domain optical coherence tomography (SD-OCT) at week 24; NVG classification at 24 weeks on a scale from 1 (non-NVG) to 6 (most advanced NVG) based on central reading of neovascularisation and incidence, causality and intensity of adverse events between the treatment arms.

All randomised subjects, regardless of treatment group, may receive rescue treatment (e.g. PRP, cryotherapy or other, as routinely performed at the clinical site) at any time during the trial if they progress to anterior segment neovascularisation (NVI or NVA), NVD, or clinically relevant NVE in the fundus. In this case, the primary endpoint is reached and the patient will not further receive study medication. Should a patient receive rescue treatment with intravitreal anti-VEGF (i.e. Lucentis®, Eylea®) because of macular oedema (individual decision of the investigator), the patient will not further receive the study drug, but will be followed up for safety reasons.

The clinical trial will be performed in consideration of the Declaration of Helsinki and the International Conference on Harmonisation – Good Clinical Practice (ICH-GCP). The protocol (Version No.:1.4/Date 7 Jan 2015 Final) is approved by the institutional Ethics Committee from the Landesärztekammer Rheinland-Pfalz, Germany, and by the French Ethics Committee. Ethics approval in other participating countries/sites will follow. Any protocol amendment will be approved by the Ethics Committees and will be distributed to all participating trial sites via the sponsor’s delegated clinical research organisation. Additionally, all necessary documents in the current version will be available on a password-protected website. Personal information of participants will be treated as confidential. Only anonymous data will be forwarded to the sponsor for scientific evaluation. Data entry and storage is performed via an electronic data capture (EDC) system, including plausibility checks.

### Statistics

Presentation of the sample size is based on both aganirsen doses combined demonstrating superiority placebo on the co-primary endpoints of neovascularisation and IOP at 24 weeks. Calculation of the sample size is based on the following: (1) An aganirsen to placebo ratio of 2:1, (2) Neovascularisation measured dichotomously (NVG = yes/NVG = no) with estimated disease rates in the population of 0.25 for aganirsen and 0.48 for placebo. Testing of neovascularisation will be done using the normal approximation to the chi-square distribution for consistency with O’Brien-Fleming Alpha spending, which is also computed based on normal approximation, (3) IOP measured dichotomously with estimated rates of events in the population of 0.24 for aganirsen and 0.41 for placebo at 24 weeks and (4) Up to four performed analyses. Based on albeit heterogeneous historical data, we estimate that 25% of subjects in the aganirsen group and 48% in the placebo group will develop NVG by 24 weeks. Given a 2:1 aganirsen to placebo assignment ratio, a total of 300 evaluable subjects – 200 with aganirsen and 100 with placebo – will provide 80% power to reject the null hypothesis that aganirsen is no better than placebo. Power computation is based on the chi-square test with a two-sided alpha = 0.05. Assuming that approximately 10% of subjects will not be evaluable due to loss to follow-up and other reasons, a total of 333 subjects are planned for this trial. Missing values of NVG and IOP will be imputed using the last observation carried forward (LOCF) method.

Primary efficacy will be assessed up to four times: two initial futility analyses, followed by an interim analysis for success/futility/continuation and a final analysis. The data will be summarised in tables listing the mean, standard deviation, minimum, median, maximum and number of subjects in a group for continuous data or in tables listing count and percentage for categorical data, where appropriate. Data listing by subject will be provided. The effects of noncompliance, dropouts and covariates, and their interactions with treatment will be assessed to determine the impact on the general applicability of results from this study. Secondary comparisons will be considered exploratory, so that there will be no Type I error adjustment for multiple secondary comparisons.

### Co-primary efficacy I: NVG

The primary efficacy analysis will compare the proportion of subjects who developed NVG in the combined aganirsen arms versus in the placebo group. The analysis will test the following hypotheses: H0: P(NVG)_aganirsen_ ≥ P(NVG)_placebo_ and H1: P(NVG)_aganirsen_ < P(NVG)_placebo_, where P(NVG) is the proportion of subjects developing NVG by 24 weeks. The hypotheses will be tested using chi-square normal approximation. (‘Yes’: development of NVI, NVA, NVD and/or NVE, or rescue treatment and ‘No’: otherwise).

### Co-primary efficacy II: IOP

H0: Proportion (IOP Failure)_aganirsen_ ≥ Proportion (IOP Failure)_placebo_ and H1: Proportion (IOP Failure)_aganirsen_ < Proportion (IOP Failure)_placebo_, where IOP Failure = increase in IOP from baseline to week 24 by ≥ 20% and IOP > 21 mmHg, or rescue treatment, and IOP success = otherwise.

### Comparison by normal approximation to chi-square test

For both co-primary endpoints: (I) At interim analysis after data of 240 subjects have accumulated using two-sided alpha = 0.02442 and (II) If needed, after data from 300 subjects have accumulated using two-sided alpha = 0.04288. Interim and final alphas determined by the O’Brien-Fleming method.

### Secondary efficacy analysis

Aganirsen arms combined versus placebo: secondary analyses will be considered exploratory. Time to development of anterior neovascularisation will be compared by *t* test or Wilcoxon rank sum test (depending on distribution), since missing data will be imputed using LOCF and there will be no censored data.

Continuous change from baseline to week 24 parameters (e.g. size of retinal nonperfusion areas) will be assessed by analysis of covariance (ANCOVA), the covariate being a subject’s baseline variable, to prevent regression towards the mean. If the model shows a lack of fit, ANCOVA will be conducted on ranked data. The number of additional laser treatments will be compared between groups by Poisson regression.

#### Imaging substudy

Because the imaging analysis is a novel investigation, its analysis will be iterative and relatively open-ended; the choice of statistical analyses is likely often to depend on the results obtained in preceding analyses. Statistical analysis of the imaging data will be performed in three parts, all using 24-week data: (1) Development of an objective scoring technique based on receiver operating characteristic curve methodology, (2) Assessment of the relationship between continuous severity scoring of the disease and imaging parameters and (3) Comparison of imaging parameters between the aganirsen exposure arms and placebo. Two thirds of the data of all arms combined will be used to develop the objective scoring methodology. This will comprise the ‘learning’ dataset. One third of the data will not be included in the initial analysis and will be retained as the ‘validation’ dataset. Objective imaging parameters (e.g. area of iris covered by neovessels) will be related to NVG diagnosis using a stepwise logistic regression. The final model will retain imaging parameters with *P* ≤ 0.10, including interactions. Each subject will receive a score generated by the final model.

#### Biomarker substudy

Statistical analysis of the tear and blood data will be performed in three parts: (1) Identifying factors that discriminate between subjects with and without NVG, (2) Analysing the longitudinal effect of treatment on immunological responses of the eye and (3) Identifying factors that predict a successful outcome of the treatment.

Because this study is exploratory, different statistical classification approaches will be used: (a) Logistic regression, including the process of variable selection and (b) Data mining tools (e.g. supervised clustering and classification trees). To optimise the model parameters, cross-validation and bootstrap methods will be applied. The longitudinal effects of aganirsen versus placebo on immunological responses of the eye will be examined. Essentially, the patterns of immunological response over time in the treatment groups will be examined. This longitudinal effect will be examined by using repeated measures analysis of variance and regression models.

#### Risk factor substudy

In this substudy, general laboratory results at baseline and subject demographics will be related to the development of NVG in three groups: (1) Study subjects who developed NVG, (2) Study subjects who did not develop NVG and (3) Age-matched controls. Differences in laboratory results will be assessed to evaluate the degree to which laboratory results and baseline factors predict the development of NVG. Additional analyses will examine the development of NVG in study subjects by whether they received active treatment. Analyses for discriminating between groups will include dichotomous and multinomial logistic regression as needed.

## Discussion

The STRONG trial is a phase II/III, prospective, randomised, placebo-controlled, double-masked, three-armed multicentre trial for the study of the topical treatment of iCRVO to prevent NVG with a newly developed eye emulsion. The study has the potential to offer a new, safe and effective treatment for patients suffering from iCRVO who have a high risk of developing NVG. NVG develops as a result of iCRVO typically after an average period of approximately 90 days but might also appear as early as 2 weeks or, alternatively, after a much longer period of time [12]. Early diagnosis of the disease followed by immediate treatment is critical to avoid a further development to neovascularisation and NVG. Early changes of the disease are present, although they are not visible in many cases [13]. Hypoxia is responsible for the release of angiogenic factors, including VEGF, resulting in growth of new vessels in the anterior chamber angle (NVA), iris surface (NVI), the optic disc (NVD) and elsewhere in the retina (NVE). IRS-1 is expressed, in particular, in human retinal pathologies, such as hypoxia, and increases VEGF expression further along in the cascade [5]. Therefore, IRS-1 has been reported to have an important role in pathological angiogenesis and retinal neovascularisation [7, 14]. Aganirsen inhibits the biosynthesis of interleukin-1β and IRS-1 in pathological angiogenesis by blocking VEGF messenger ribonucleic acid to restore normal VEGF expression, thus preventing neovascularisation and NVG [6, 7].

According to their mechanism of action, anti-VEGF agents have also been considered in the management of neovascularisation in iCRVO, but they are used off-label [15–19] and usually in a late stage when neovascularisation has already occurred. In addition to the patients’ burden, several ocular drawbacks and side effects such as IOP elevation, endophthalmitis, retinal detachment or injury of the lens [20, 21], might be associated with the invasive and often multiple intravitreal injections. At present, PRP is the common method used for reducing retinal ischaemia or neovascularisation in patients with iCRVO, even though there are major drawbacks of PRP, such as persistent visual field defects. To target the retina with aganirsen and avoid the invasive route of intravitreal injections, an emulsion formulation of the active substance aganirsen was developed by Gene Signal SA (Genopole Biopark, Evry (Paris), France). Despite advances in the medical and surgical management of glaucoma, the visual prognosis for patients with NVG remains poor [22]. Furthermore, the current therapeutic treatment options show many disadvantages including peripheral visual field loss after PRP [23], limitations and discomfort.

The study drug aganirsen is geared toward early treatment of the underlying disease, iCRVO, to avoid the progression to neovascularisations and further NVG. Aganirsen was developed as an eye emulsion to reach the affected areas of the retina and is applied topically. It is hoped that the topical application of aganirsen eye emulsion will have fewer side effects compared to the current standard therapy, which includes laser therapy, intravitreal injections and/or surgery. It could, therefore, overcome the disadvantages and limitations of the current therapeutic options, particularly the surgical ones, and has the potential to change the current management of NVG progressing to iCRVO because it may prevent NVG instead of treating its symptoms. The outcome of the trial will have potentially significant economic impacts considering the high costs for the health care system due to advanced glaucoma and the risk of blindness. Furthermore, if successful, it will have a significant impact on the patients’ QoL, considering the painful end stages of NVG, risks of laser therapy, possible blindness and enucleation, etc. Positive study results of this EMA-approved study protocol will pave the way to marketing authorisation in Europe.

The STRONG study, including its substudies, will provide important data on the natural course of iCRVO and NVG and its risk factors. One approach to better understand the natural course of the disease is a semiautomatic method for vessel quantification on photographs [8, 24], which will allow for the evaluation and grading of the development of NVI and NVA and the quantification of iris vessel dilation and the degree of NVI. Furthermore, proteomics and immunoproteomics of biomarkers in the blood and tear fluid have been shown to be useful in improving the diagnostics and prognosis of different ophthalmologic diseases and understanding their pathogenesis [25–32]. Prognostic parameters for treatment effects could also be established [27, 33–38]. In addition to the well-known classical risk factors, new haemostasis-related ones have been investigated in patients affected by CRVO [39]. There is one study suggesting that high levels of soluble endothelial protein C receptor (sEPCR) might be a risk factor for retinal vein occlusion (RVO) because the levels were higher in patients with CRVO than in those with branch retinal vein occlusion (BRVO) and controls [40]. Together with the biomarker analysis and the identification of patients at a high risk for the development of NVG after iCRVO, there could be a great potential for the early diagnosis of patients who will convert to NVG after iCRVO, which would allow for an intervention before the progression to neovascularisation to avoid progression to NVG.

The pool of patients evaluated by the STRONG consortium is small for several reasons. First, iCRVO is a rare disease (EMA Orphan Drug Designation EMA/COMP/256922/2014). The prevalence of CRVO is between 2.8/10,000 and 4.2/10,000 [41]. The ischaemic form of CRVO represents 20% [42] to 33% [11], which results in a prevalence of approximately 1/10,000 for people aged 30 years or older (well below the EMA’s orphan ceiling of 5/10,000). Additionally, the inclusion and exclusion criteria are very strict but are in line with the EMA Protocol Assistance. The criteria will ensure the identification of patients with iCRVO with a very high risk of developing NVG [2, 10, 43, 44]. However, patients with iCRVO not only have a high risk for the development of NVG, but they also frequently suffer from macular oedema, which is now frequently treated with anti-VEGF agents, such as off-label bevacizumab, or with ranibizumab (Lucentis®) or aflibercept (Eylea®) after marketing authorisation for the treatment of macular oedema after CRVO [22, 45]. The treatment regimen for macular oedema following CRVO has changed in the last few years, and macular oedema is now often treated at a very early stage. Therefore, patient recruitment is becoming increasingly difficult. Patients with previous anti-VEGF therapy (45 or 90 days before screening, depending therapy type) are excluded from this trial. However, ethical concerns for patients can be excluded because rescue treatment with anti-VEGF for macular oedema is allowed at any time if deemed necessary (individual decision of the investigator). Following the guidelines of the German Ophthalmological Society, for patients with a visual acuity <0.05, an injection should only be administered if there remains, due to the morphological findings, a legitimate prospect of a visual improvement by the therapy to more than 0.05 [46]. Only patients with a very bad visual acuity (<0.1) with a poor prognosis will be enrolled. In addition, there is the possibility of improvement of the macular oedema (delaying or avoiding the necessity of an anti-VEGF treatment) by aganirsen treatment in two thirds of all patients. PRP, and also cryotherapy, is strongly advocated and practiced widely for the management of iCRVO if neovessels have already formed [10, 11]. The lack of regulatory-approved preventive therapies for NVG necessitates the comparison to a placebo. The treatment of NVG with anti-VEGF is off-label [44]. If rescue treatment for macular oedema is necessary, the patient will not continue to receive the study drug but will be followed up for safety. The trial patients will not incur any disadvantages in participating in the trial, even if they are allocated to the placebo arm because currently, there is no therapy available to prevent NVG resulting from iCRVO. The close visit scheme will further help to detect progression of the disease and to intervene quickly.

ICRVO and NVG are both rare diseases [2, 11, 41, 42]. Aganirsen received Orphan Medicinal Product status for the treatment of NVG in 2003 (EU/3/03/161) and Orphan Drug status for the treatment of CRVO in 2014 (EMA/COMP/164746/2014; EMA/OD/008/14). The recruitment period is quite short for a rare disease, which will also make it difficult to reach the recruitment target. To overcome this challenge, a project partner, AIBILI, the coordinator of the EVICR.net, is in close contact with all of the member sites across several European countries to ensure fast evaluation of potential trial sites and recruitment of patients.

The fact that aganirsen will be applied topically by the patients themselves could influence the results and outcomes of the study. Glaucoma therapy with topical medications requires good patient compliance to achieve optimal therapeutic results. A consistent and correct application of the drug is difficult, especially for older patients. Patients might forget to apply the medication or they might not be aware of its importance due to the asymptomatic course of the disease [47–49]. Studies have shown a rate of noncompliance in glaucoma patients of approximately 25-50%. Compliance is also strictly required for the STRONG trial. Therefore, several methods will be applied to increase and control patient compliance, such as drug accountability after drug return and evaluation of patient diaries. The patient advocacy group Bundesverband Glaukom-Selbsthilfe e.V., a consortium member, supports the important instruction of patients regarding this chronic disease and the correct eye drop application to overcome this matter.

The STRONG trial has to overcome several difficulties, but if successful, offers many therapeutic chances and opportunities for patients with this rare disease.

### Trial status

The study is in preparation. It is not yet open for participant recruitment.

## Additional file

Additional file 1: SPIRIT Checklist. (DOC 131 kb)

## Abbreviations

(i)CRVO: (Ischaemic) central retinal vein occlusion
(s)EPCR: (Soluble) endothelial protein C receptor
(SD-)OCT: (Spectral domain) optical coherence tomography
ANCOVA: Analysis of covariance
BCVA: Best-corrected visual acuity
BRVO: Branch retinal vein occlusion
EMA: European Medicines Agency
EQ-5D: EuroQoL five dimensions questionnaire
ETDRS: Early Treatment Diabetic Retinopathy Study
ICH-GCP: International Conference on Harmonisation – Good Clinical Practice
IOP: Intraocular pressure
IRS-1: Insulin receptor substrate-1
LOCF: Last observation carried forward
NEI-VFQ-25: National Eye Institute Visual Function questionnaire 25
NVA: Neovascularisation of the angle
NVD: Neovascularisation of the disc
NVE: Neovascularisation elsewhere
NVG: Neovascular glaucoma
NVI: Neovascularisation of the iris
PRP: Pan-retinal photocoagulation
QoL: Quality of life
RVO: Retinal vein occlusion
TNF: Tumour necrosis factor
VEGF: Vascular endothelial growth factor
